# Supplementary material for: Development of Hydroxamic Acid Compounds for Inhibition of Metallo-β-Lactamase from Bacillus anthracis
Source: Int J Mol Sci. 2022 Aug 15;23(16):9163. doi: 10.3390/ijms23169163 (PMC9408887; doi:10.3390/ijms23169163)
Supplement: Supplementary file 1 [file ijms-23-09163-s001.zip › ijms-1806639-supplementary.pdf]

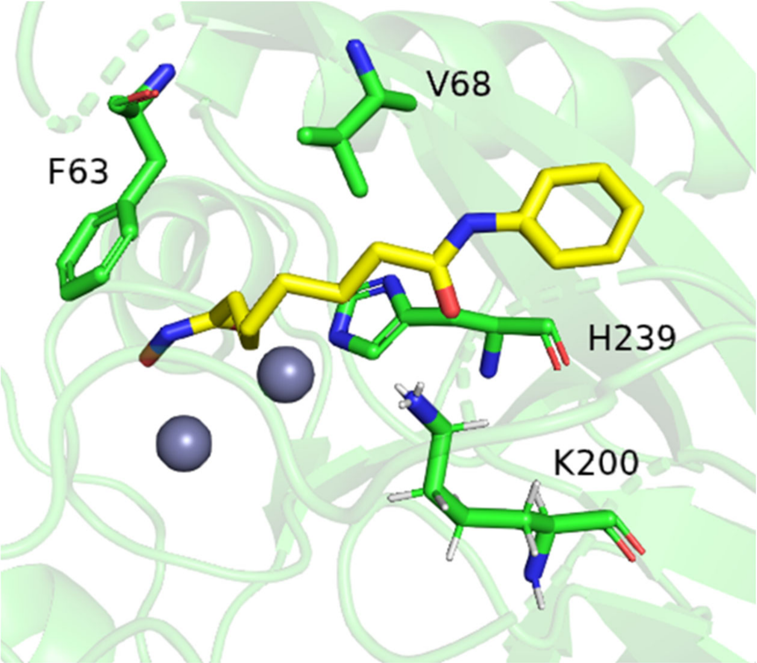

Figure S1. Average atomic coordinates of the root mean square fluctuation of SAHA. .

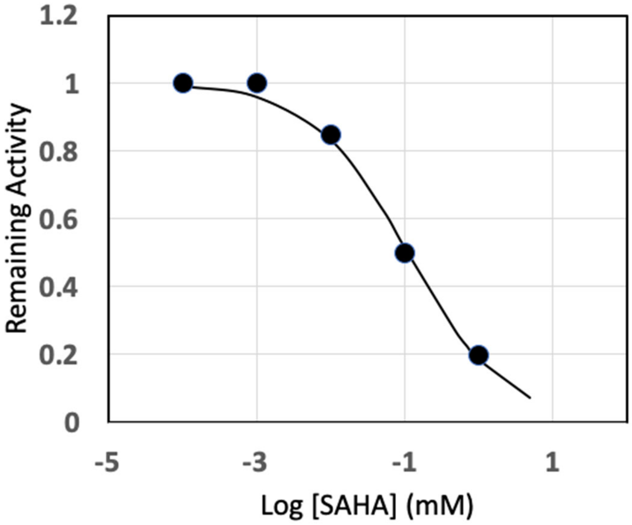

Figure S2. The inhibition of Bla2 by SAHA.

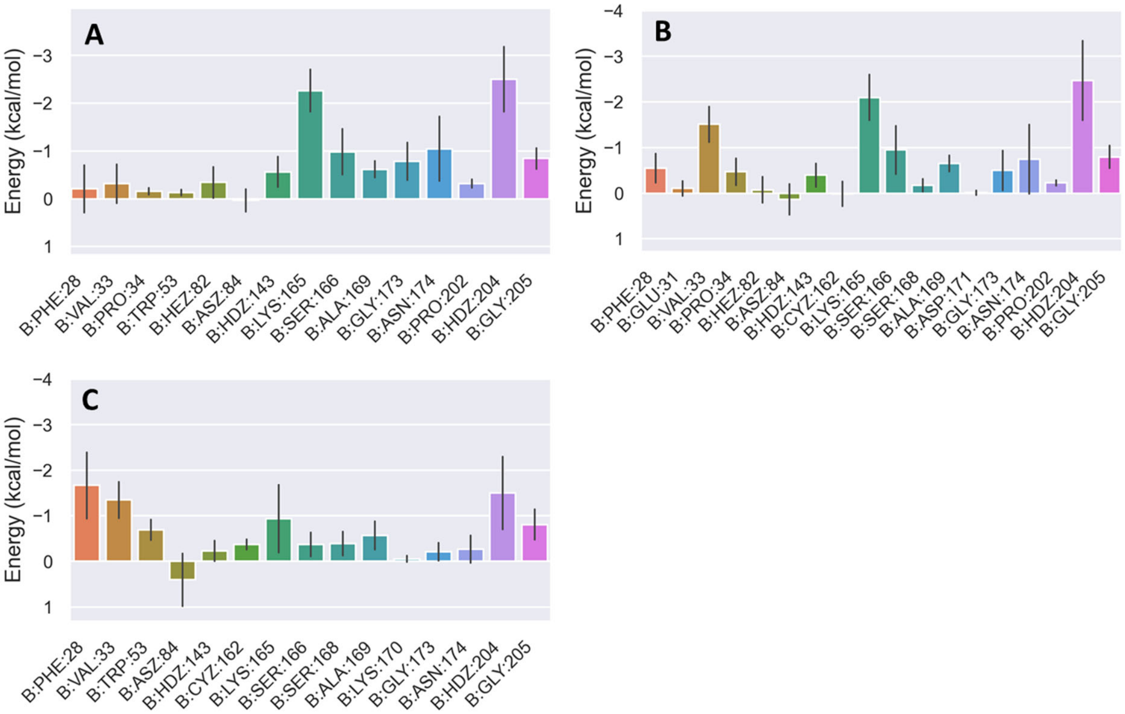

Figure S3. Per-Residue Decomposition figures of the (A) Compound 4 simulation, (B) Compound 6 simulation, and (C) SAHA simulation. Residue sequence numbering is shortened by 35, in relation to residue references in the rest of the paper.

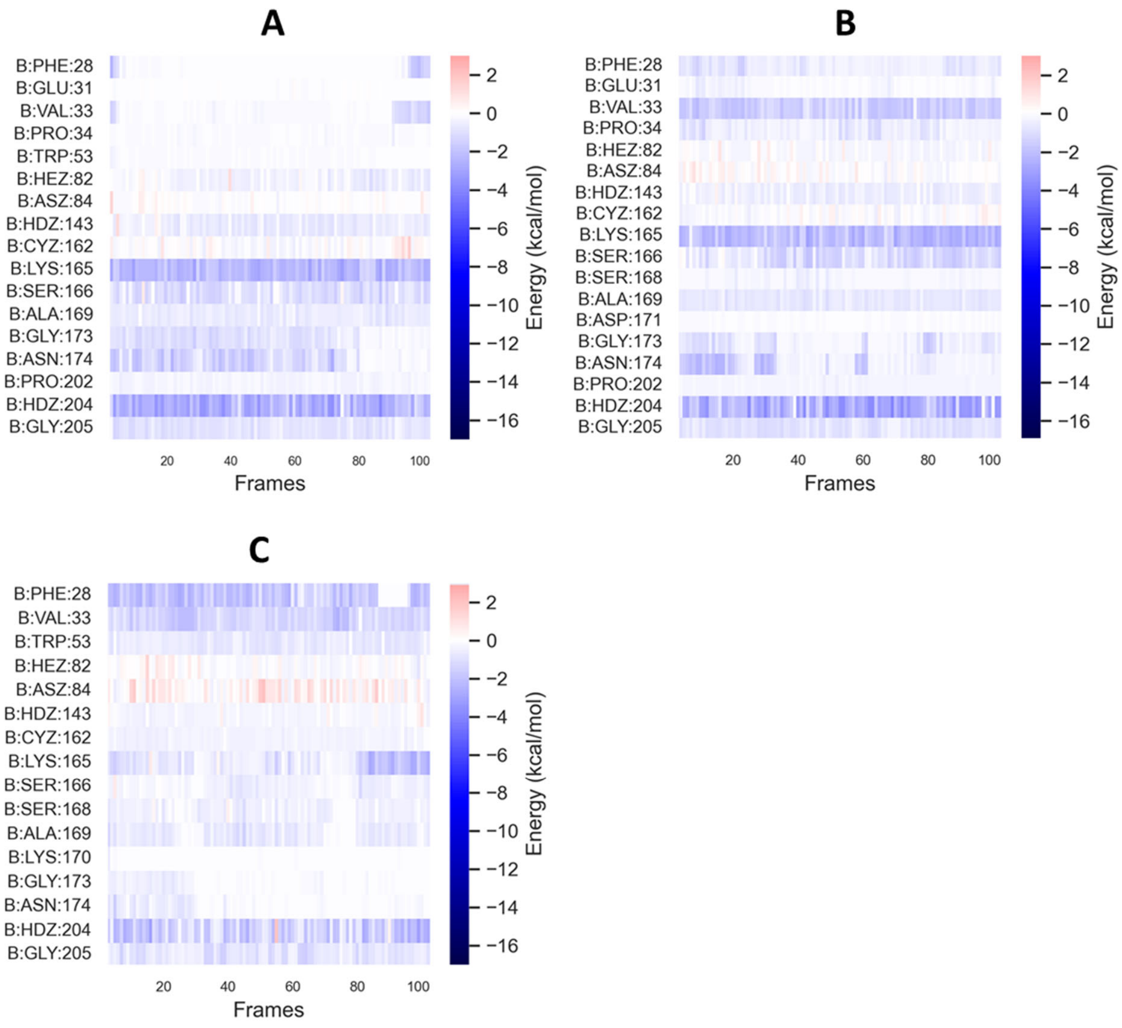

Figure S4. Per-Residue Decomposition Heatmap of (A) Compound 4 simulation free energy, (B) Compound 6 simulation free energy, and (C) SAHA simulation free energy. Residue sequence numbering is shortened by 35, in relation to residue references in the rest of the paper.

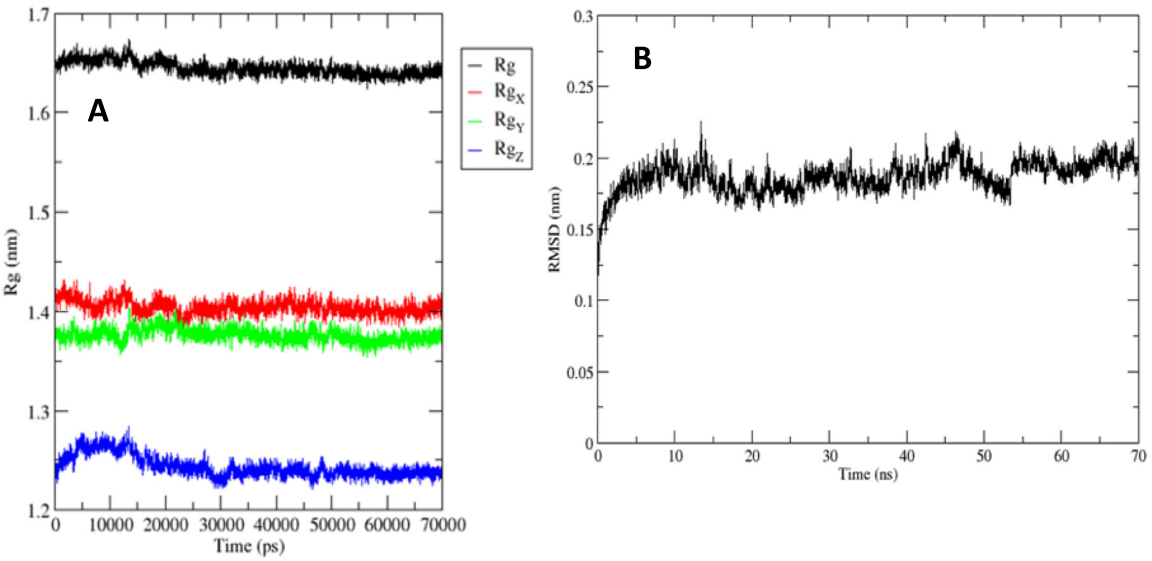

Figure S5. (A) The radius of gyration of the protein backbone in the 70 ns simulation. (B) The RMSD of the protein backbone in the 70 ns simulation.
